# Roles of Astrocytic Endothelin ET_B_ Receptor in Traumatic Brain Injury

**DOI:** 10.3390/cells12050719

**Published:** 2023-02-24

**Authors:** Shotaro Michinaga, Shigeru Hishinuma, Yutaka Koyama

**Affiliations:** 1 Department of Pharmacodynamics, Meiji Pharmaceutical University, 2-522-1 Noshio, Tokyo 204-8588, Japan; 2 Laboratory of Pharmacology, Kobe Pharmaceutical University, 4-19-1 Motoyama-Kita Higashinada, Kobe 668-8558, Japan

**Keywords:** astrocyte, traumatic brain injury, endothelin, ET_B_ receptor, blood–brain barrier, neuroinflammation

## Abstract

Traumatic brain injury (TBI) is an intracranial injury caused by accidents, falls, or sports. The production of endothelins (ETs) is increased in the injured brain. ET receptors are classified into distinct types, including ET_A_ receptor (ET_A_-R) and ET_B_ receptor (ET_B_-R). ET_B_-R is highly expressed in reactive astrocytes and upregulated by TBI. Activation of astrocytic ET_B_-R promotes conversion to reactive astrocytes and the production of astrocyte-derived bioactive factors, including vascular permeability regulators and cytokines, which cause blood–brain barrier (BBB) disruption, brain edema, and neuroinflammation in the acute phase of TBI. ET_B_-R antagonists alleviate BBB disruption and brain edema in animal models of TBI. The activation of astrocytic ET_B_ receptors also enhances the production of various neurotrophic factors. These astrocyte-derived neurotrophic factors promote the repair of the damaged nervous system in the recovery phase of patients with TBI. Thus, astrocytic ET_B_-R is expected to be a promising drug target for TBI in both the acute and recovery phases. This article reviews recent observations on the role of astrocytic ET_B_ receptors in TBI.

## 1. Introduction

Traumatic brain injury (TBI) is critical damage to the brain caused by a sudden insult, such as traffic accidents, falls, collisions, and sporting activities. TBI is a major cause of death and disability worldwide. Even in surviving patients, TBI causes severe sequelae in motor, sensory, mental, and cognitive functions, resulting in decreased quality of life (QOL). Therefore, much effort has been devoted to realizing effective therapies for TBI, that is, treatments to protect the brain from damage in the acute phase and promote the recovery of neurological function in TBI patients with sequelae.

Brain damage caused by TBI is classified as primary or secondary [1,2]. Primary damage includes direct physical injury to the brain parenchyma, such as skull fractures, intracranial hemorrhage, compression/deformation of nerve tissue, diffuse axonal injury, and crushing of blood vessels. Biochemical, cellular, and physiological alterations induced by primary damage propagate from the impact core to the peripheral area and aggravate brain injury (secondary damage) [1,2]. Pathological events that induce secondary damage in TBI around the impact core include excitotoxicity, cerebral hypoperfusion, brain edema, and neuroinflammation. While primary damage is irreversible and difficult to reduce, secondary damage is partly reversible and remediable. Therefore, therapies for TBI in the acute phase focus on reducing secondary damage. Current treatments for the acute phase of TBI include decompressive craniotomy, hyperosmolar treatment, barbiturates, sedation, and hypothermia therapy. However, these treatments are insufficient and may have adverse effects in some cases. Several candidate drugs have shown beneficial effects in preclinical studies using experimental TBI animal models, but clinical trials have failed to show significant effects in patients with TBI [3,4,5]. In addition, for TBI patients with sequelae, treatments to promote the recovery of neurological functions impaired by TBI are required, which are currently performed by physical therapy. Although many studies have shown that physical therapy promotes synaptic regeneration in damaged nervous systems [6,7,8], no medication is clinically used to enhance its efficiency. Therefore, research and development of medicines applied in the acute and recovery phases of TBI have been extensively conducted.

Many studies have clarified the roles of astrocytes in nerve damage and recovery processes in several brain disorders, including TBI [9,10,11]. Based on these studies, the regulation of astrocytic functions has been proposed as a novel therapeutic strategy for TBI. Endothelin (ET) is one of the factors regulating the pathophysiological functions of astrocytes in damaged nerve tissues [12]. ET receptor signaling-mediated pathophysiological reactions include ischemia, neuropathic pain, and disruption of the blood–brain barrier (BBB) [12]. We previously observed that ET_B_ receptor (ET_B_-R) but not ET_A_ receptor (ET_A_-R) was highly distributed in astrocytes and that an ET_B_-R antagonist but not an ET_A_-R antagonist alleviated pathological conditions, including the proliferation of reactive astrocytes, BBB disruption, and brain edema in TBI model mice [13]. In this review, the roles of the ET_B_ receptor (ET_B_-R) in astrocytic functions in TBI are reviewed. In addition, the possibility of astrocytic ET_B_-R as a novel drug target for TBI is discussed.

## 2. Pathophysiological Responses of Astrocytes to TBI

**Figure 1:**
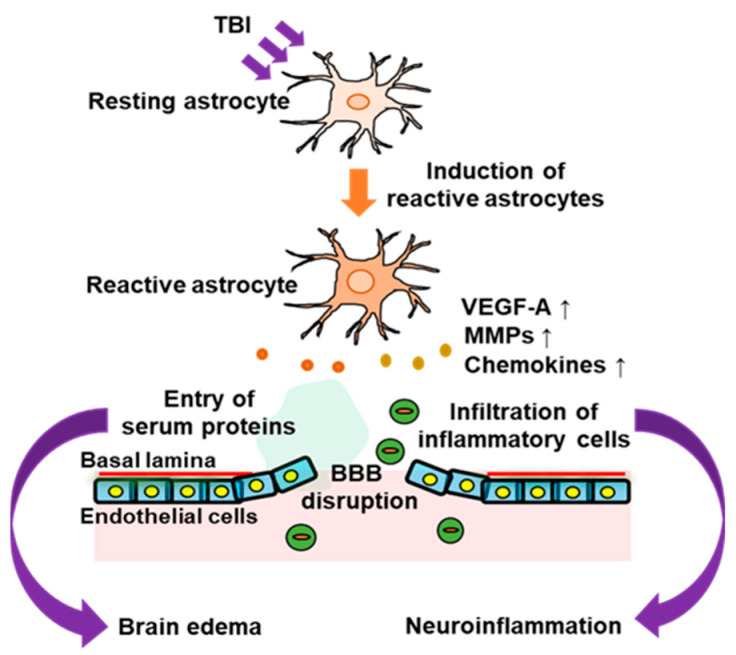
Involvement of astrocytes in BBB disruption in the acute phase of traumatic brain injury (TBI). In the acute phase of TBI, activation of astrocytes occurs around the damaged brain area. The astrocytic activation is accompanied by increased expressions of astrocyte-derived vascular permeability factors, such as vascular endothelial growth factor-A (VEGF-A), matrix metalloproteinase 9 (MMP9), and chemokines. This impairs the barrier function of the blood–brain barrier (BBB) and allows entry of inflammatory cells and serum proteins into the brain parenchyma, which leads to neuroinflammation and brain edema.

In response to brain disorders, astrocytes change their phenotype to that of reactive astrocytes, which are characterized by increased glial fibrillary acidic protein (GFAP) expression and hypertrophy. Reactive astrocytes are involved in the progression of many brain pathologies and the regeneration of the injured nervous system. In patients with TBI, phenotypic conversion to reactive astrocytes is predominantly observed in damaged areas [14]. Similarly, reactive astrocytes were also increased in experimental TBI model animals [13,14,15,16]. Brain edema occurs during the acute phase of TBI. Increased intracranial pressure accompanied by brain edema causes impairment of the nervous system and often results in the death of patients with TBI. In addition, neuroinflammation in the acute phase exacerbates neuronal damage caused by TBI and causes various neurological dysfunctions in patients affecting motor, sensory, and cognitive activities. Disruption of the BBB underlies the development of brain edema and neuroinflammation caused by TBI. That is, the hyperpermeability of brain microvascular endothelial cells, which constitute the BBB, can allow the infiltration of inflammatory cells and serum proteins into the cerebral parenchyma damaged by TBI. Astrocytes support the integrity of the BBB, and their end feet surround a large part of the basolateral side of brain microvessels. The permeability of brain microvascular endothelial cells responsible for the BBB is regulated by their interaction with astrocytes. Functional alterations in astrocytes in TBI lead to excessive hyperpermeability of the BBB, which allows the entry of inflammatory cells and serum proteins (Figure 1).

Increased production of various cytokines and chemokines in the acute phase of TBI has been reported [17,18]. The production of astrocytic cytokines and chemokines is stimulated by several signaling molecules released from damaged cells [19,20,21]. In TBI, astrocytic IL-33 is increased in the human and mouse brain and promotes the accumulation of microglia/macrophages at the site of injury [22]. Xue et al. [23] showed that astrocytes produce C-C Motif Chemokine Ligand 7 (CCL7), which promotes microglia-mediated inflammation in a TBI rat model. Other astrocytic vascular permeability regulators, such as matrix metalloproteinase 9 (MMP9) and vascular endothelial growth factor-A (VEGF-A), are also upregulated by TBI and cause disruption of the BBB [13,24,25,26,27]. These results indicate that astrocytes have a detrimental effect on BBB function during the acute phase of TBI. However, some reports suggest that astrocytes have supporting roles in BBB function, by which brain edema and neuroinflammation in TBI are reduced. Hu et al. [28] showed that the ablation of astrocytes exacerbated the infiltration of monocytes into the cerebral parenchyma and neuronal loss in mice with brain stab injuries. Gao et al. [29] found that programmed cell death 1 (PD-L1) signaling in reactive astrocytes prevented excessive neuroimmune and neuronal damage in a controlled cortical impact-induced TBI mouse model. Astrocyte-derived exosomes also protect hippocampal neurons by suppressing mitochondrial oxidative stress and apoptosis in rats with TBI [30]. We also found that astrocyte-derived vascular protective factors, such as angiopoietin-1 (ANG-1) and sonic hedgehog (SHH), were increased in TBI model mice [31,32]. These findings suggest that astrocytes suppress TBI-induced neuroinflammation and BBB disruption and exert protective actions against neuronal damage in TBI. Additionally, expression levels of MMP-9 and VEGF-A increased at 6 h to 5 days after TBI, whereas expression levels of ANG-1 and SHH increased at 3 to 10 days after TBI [13,31,32]. This finding shows a possibility that conversion to reactive astrocytes enhances both detrimental and supportive actions on BBB function depending on the phase of TBI.

During the recovery phase of TBI, new synapses are formed in damaged areas, which are supported by neurogenesis from neural progenitors and axonal elongation [33]. This remodeling of the damaged nervous system is the mechanism that underlies the recovery of brain functions impaired by TBI. Some astrocyte-derived factors have been shown to promote the remodeling of the nervous system damaged by TBI. Astrocyte-produced apolipoprotein E [34] and S100b [35] promote neurogenesis and recovery of cognitive function impairments in TBI. Thrombospondins (TSPs) are astrocyte-secreted proteins that promote synaptogenesis [36,37]. Cheng et al. reported that TSP-1 was increased in TBI and that TSP-1 knockout mice exhibited significantly worse neurological deficits in motor and cognitive functions [38]. Production of neurotrophin family neurotrophic factors is upregulated by TBI and promotes neuroprotection in the acute phase, as well as regeneration in the recovery phase [39]. Astrocytes are the major source of nerve growth factor (NGF), which is also upregulated by TBI [40]. Administration of NGF to the rat brain reversed the decrease in cholinergic nerves induced by TBI and enhanced cognitive function [41]. Treatment to increase the production of brain-derived neurotrophic factor (BDNF) in astrocytes restored neuronal function impaired by TBI [42,43]. Hao et al. showed that exogenous neurotrophin-3 (NT-3) administration to TBI model rats promoted neural stem cell proliferation and synaptogenesis [44]. These findings indicate that the ability of astrocytes to produce neurotrophic factors is beneficial in promoting nerve regeneration during the recovery phase of TBI.

## 3. Endothelin in TBI

### 3.1. Endothelin Receptor Signal Pathways and Pathophysiological Reactions

**Figure 2:**
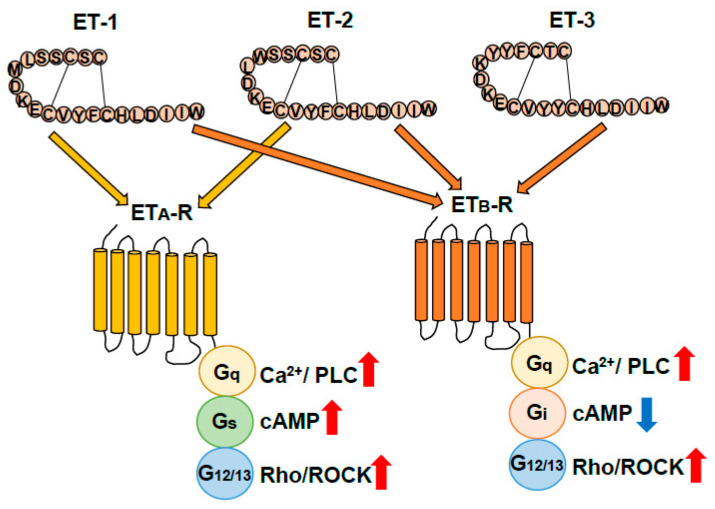
Classification of endothelin receptors. Endothelins (ETs) exert their physiological actions through two G-protein-coupled receptor subtypes, ET_A_ (ET_A_-R) and ET_B_ receptors (ET_B_-R). ET_A_ receptor signal pathways mediate Gq, Gs, and G_12/13_ resulting in activation of Ca^2+^/phospholipase C (PLC), cAMP, and G_12/13_/Rho/Rock signal pathways. ET_B_-R signal pathways mediate Gq, Gi, and G_12/13_ resulting in activation of Ca^2+^/PLC and Rho/Rock signal pathways and suppression of cAMP signaling pathways [12].

The ET ligand family, ET-1, ET-2, and ET-3, was initially discovered as vasoconstrictor peptides produced by vascular endothelial cells. ET-1 is present in the brain and is one of the factors involved in pathophysiological responses of damaged nerve tissues [45,46]. ET-2 is largely limited to the gastrointestinal tract, sex organs, and pituitary gland, and ET-3 is abundantly expressed in the intestine, pituitary gland, and brain [12]. Receptors for ETs, which are seven-transmembrane G-protein-coupled receptors, are classified into two distinct types: ET_A_-R and ET_B_-R, which are encoded by the EDNRA and EDNRB genes, respectively (Figure 2). ET_A_-R shows higher affinities for ET-1 and ET-2 than for ET-3, whereas ET_B_-R has an equal affinity for all three ET ligands. Both ET_A_-R and ET_B_-R are linked to the Gq protein and increase intracellular Ca^2+^ by activating phospholipase C (PLC). However, ET_A_-R and ET_B_-R have different regulatory mechanisms for adenylate cyclase-mediated signals. ET_A_-R is Gs-linked to increase cAMP, whereas ET_B_-R is linked to Gi and suppresses the signal (Figure 2). Both ET_A_-R and ET_B_-R are also linked to the G_12/13_ protein (Figure 2). The G_12/13_ protein-mediated signal activates the Rho protein, a low molecular weight G-protein, and stimulates Rho-associated protein kinase (ROCK), which regulates cellular proliferation, Ca^2+^, and cytoskeletal actin reorganization [12].

Several studies imply that ET-R-mediated calcium-dependent signaling contributes to neuroinflammation. ET-1 increases intracellular Ca^2+^ by the influx of calcium and release of intracellular calcium stores. ET_A_-R-mediated calcium-dependent responses include activation and degranulation of neutrophils by calcium influx [47]. ET_B_-R-mediated calcium-dependent responses include the chemotactic migration of neutrophils by the release of intracellular calcium [47]. In addition, astrocytic dysregulation of Ca^2+^ homeostasis promotes the release of inflammatory factors, which cause neuroinflammation in Alzheimer’s disease [48]. Because ET_B_-R is highly expressed in astrocytes and ET_B_-R signaling promotes the production of astrocytic bioactive factors [12,13], ET_B_-R signaling may be involved in neuroinflammation by astrocytic dysregulation of Ca^2+^ homeostasis. These observations suggest that activation of ET-Rs and neuroinflammation are correlated to calcium homeostasis dysregulation.

The pathophysiological roles of ETs in the cardiovascular system, such as arterial hypertension, myocardial infarction, preeclampsia, and coronary atherosclerosis, have been well investigated. In the clinical state, several ET-R antagonists, including bosentan (non-selective), ambrisentan (ET_A_-R selective), and macitentan (non-selective), have been applied as therapeutic drugs for pulmonary arterial hypertension. ET antagonists are also expected to be therapeutic drugs for cardiovascular and other disorders [49]. In patients with several neurodegenerative disorders, cerebral ischemia, and subarachnoid hemorrhage, expression levels of ETs are increased [50,51,52,53]. Some studies showed that in addition to vascular endothelial cells, astrocytes are also a primary source of ET-1. The expression level of astrocytic ET-1 is increased by several factors, including cytokines, hypoxia, and ET-1 itself [54,55,56]. 

### 3.2. Relationships of Endothelin and TBI

Brain ET-1 production was found to be increased by TBI, whereas productions of ET-2 and ET-3 have not been investigated. Studies in patients with TBI and experimental animal models indicate that increases in brain ET-1 are closely related to the pathological conditions of TBI. Maier et al. [57] showed that ET-1 is increased in the plasma and cerebrospinal fluid of patients with TBI. Chen et al. [58] also found that ET-1 levels were significantly higher in the severe TBI group than in the mild/moderate TBI and control groups. Additionally, ET-1 is also increased in the cerebrospinal fluid and is associated with unfavorable outcomes in children after severe TBI [59]. In experimental TBI models, ET-1 levels are increased in the brain [14,27,56,60,61]. Histological observations have shown that reactive astrocytes produce ET-1 in TBI [56]. An examination using cultured cells showed that a direct physical effect on astrocytes is partially involved in TBI-induced astrocytic ET-1 production [31]. TBI causes cerebral circulation dysfunction, such as vasospasm and hyperpermeability of brain microvessels, which aggravates secondary damage [62]. Experimental animal models of cerebral ischemia and subarachnoid hemorrhage have shown that increases in brain ET-1 cause vasospasm through ET_A_-R [63,64]. ET_A_-R mediates vasospasm through protein kinase C, whereas ET_B_-R mediates vasodilation through nitric oxide synthesis [64]. These experimental results suggest that antagonism of ET_A_-R may also alleviate vasospasm in the brain damaged by TBI. 

**Figure 3:**
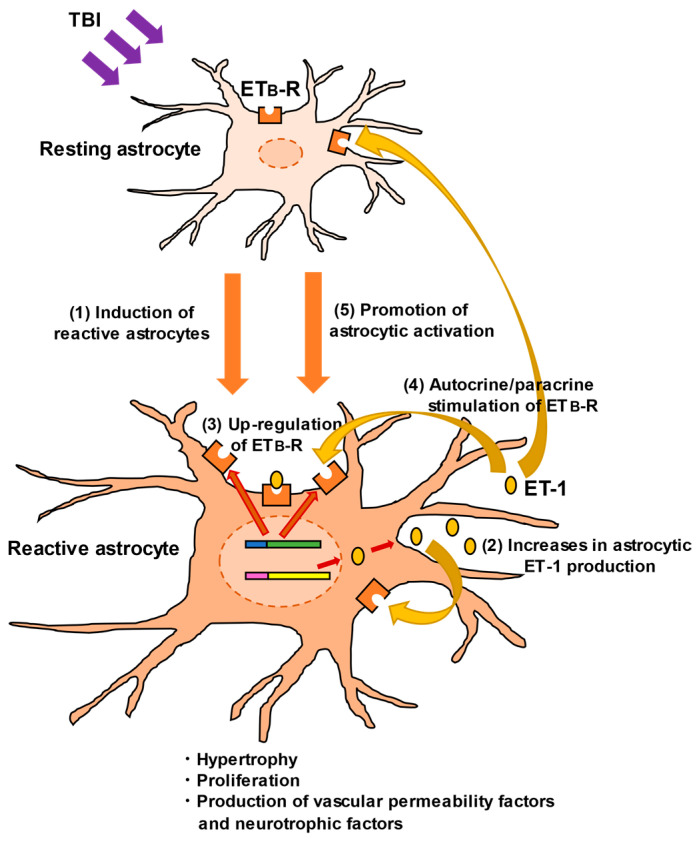
Promotion of astrocytic activation through ET-1/ET_B_-R signaling. In TBI, ET-1 induces reactive astrocytes through the following mechanisms: (1) TBI promotes the conversion of resting astrocytes to reactive ones. (2) ET-1 is produced and released from reactive astrocytes. The physical impact of TBI directly stimulates ET-1 production, although the mechanism is unclear [27,31]. (3) As well as ET-1, expression of astrocytic ET_B_-R is upregulated, accompanied by astrocytic activation. (4) Astrocyte-derived ET-1 stimulates ET_B_-R through an autocrine/paracrine mechanism. (5) ET_B_-R stimulation further promotes conversion to reactive astrocytes and enhances their functions.

ET_B_-Rs are expressed in astrocytes and brain vascular endothelial cells [56,65,66,67,68], whereas ET_A_-Rs are highly expressed in brain blood vessels but not in astrocytes [13]. Expression of astrocytic ET_B_-Rs is also upregulated in response to brain disorders, as well as astrocytic ET-1 [67,68,69]. We found that ET_B_-R expression in reactive astrocytes was significantly increased in the injured cerebrum of TBI mice [56]. The concomitant increases in both astrocytic ET ligands and receptors suggest an autocrine/paracrine mechanism mediated by ET-1/ET_B_-R signaling, which becomes more prominent in the regulation of astrocytic pathophysiological actions in TBI (Figure 3). Therefore, the role of ET_B_-R-mediated signaling in astrocytic pathophysiological responses has been investigated.

## 4. Regulation of Astrocytic Functions by ET_B_-R

### 4.1. ET_B_-R-Mediated Conversion to Reactive Astrocytes

Conversion of resting astrocytes to reactive phenotypes in response to brain injury is characterized by increased GFAP expression and hypertrophy. Hyperplasia of reactive astrocytes causes glial scar formation in injured areas, which prevents axonal elongation during the recovery of damaged nerve tissues. ET-1 is one of the factors that induce conversion to reactive astrocytes in injured nerve tissues. Intracerebroventricular administration of Ala^1,3,11,15^-ET-1, a selective ET_B_-R agonist, promotes the reactive conversion of astrocytes in the rat brain [65]. In experimental brain injury models, inhibition of ET_B_-R decreases the number of reactive astrocytes [69,70,71,72]. We also found that BQ788, a selective ET_B_-R antagonist, reduced the induction of reactive astrocytes in TBI model mice [13].

### 4.2. ET_B_-R-Mediated Proliferation Signal Pathways in Astrocytes

**Figure 4:**
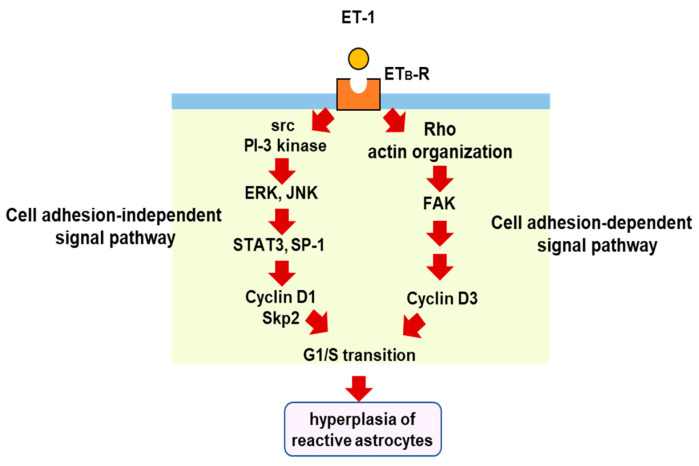
Proliferation signal pathways triggered by astrocytic ET_B_-R. ET_B_-R-mediated intracellular signals for astrocytic proliferation include cell adhesion-dependent and -independent pathways. In the cell adhesion-dependent pathway, stimulation of ET_B_-R causes cytoskeletal actin reorganization through a small G-protein, rho. The formation of focal adhesions accompanied by cytoskeletal reorganization activates FAK and increases cyclinD3 expression. In the cell adhesion-independent signal pathway, stimulation of ET_B_-R increases the activities of STAT3 and SP-1 through ERK and JNK. These transcription factors increase the expressions of cyclinD1 and S-phase kinase-associated protein 2 (Skp-2). The upregulation of these cell cycle regulatory proteins by ET-1 induces the proliferation of reactive astrocytes in brain disorders, including TBI.

The proliferation of reactive astrocytes is regulated by multiple intracellular signaling pathways. Several studies have suggested that signal transducer and activator of transcription 3 (STAT3) is a key transcription factor for astrocytic proliferation in brain injury [73,74,75]. Selective nuclear localization of phosphorylated STAT3 in reactive astrocytes was observed in the cerebrum of TBI model rats [76]. We also found that phosphorylated STAT3 increased in the cerebrum of mice with TBI [77]. In cultured astrocytes, ET-1 promotes STAT3 activation through ET_B_-R, and inhibition of STAT3 reduces cell proliferation [77], suggesting that STAT3 is involved in ET-induced astrocytic proliferation. Transcriptional target molecules of STAT3 include cyclin D1 and S-phase kinase-associated protein 2 (Skp-2), cell cycle regulators that stimulate the G1/S transition [77,78]. Histological observations in brain injury models have shown that cyclin D1 is expressed in reactive astrocytes [79]. The increase in cyclin D1 expression mediates astrocytic proliferation triggered by ET_B_-R/STAT3 signaling [77]. Other transcription factors than STAT3 are also involved in astrocytic cyclin D1 expression. We found that ET-1-activated specificity protein-1 (SP-1) promotes the expression of cyclin D1 and the proliferation of cultured astrocytes through ET_B_-R [80]. ET-1-induced SP-1 activation and cyclin D1 expression were suppressed by inhibitors of mitogen-activated protein kinases (MAPKs) [80]. Gadea et al. [71] also showed that ET_B_-R-mediated astrocyte proliferation and activation occur through the activation of ERK- and JNK-dependent pathways. ET-induced astrocytic proliferation is coordinately regulated by both cell adhesion-dependent and independent pathways under ET_B_-R activation [81]. The expression of cyclin D1 through the ERK and JNK pathways is in a cell adhesion-independent pathway, whereas astrocytic cyclin D3, another G1/S cyclin, is increased by the stimulation of ET_B_-R in a cell adhesion-dependent manner. In the adhesion-dependent mechanism, a small G-protein (Rho) and focal adhesion kinase (FAK) mediate the cell proliferation signal from ET_B_-R to cyclin D3 [82]. This diversity of proliferation pathways characterizes the ET_B_-R signal in hyperplasia of reactive astrocytes, leading to glial scar formation (Figure 4).

### 4.3. ET_B_-R- Mediated Production of Bioactive Factors in Astrocytes

In the damaged brain, reactive astrocytes produce multiple bioactive factors that increase the permeability of microvascular endothelial cells. The production of these factors disrupts the BBB, which leads to brain edema and infiltration of inflammatory cells. Activation of ET_B_-R in cultured astrocytes and rat brains stimulated the production of MMP-9, MMP-2, VEGF-A, and stromelysin-1 [83,84,85,86,87], which increases vascular permeability. At brain injury sites, activated astrocytes produce monocyte chemoattractant protein-1 (MCP-1)/C-C motif chemokine 2 (CCL2) and cytokine-induced neutrophil chemoattractant-1 (CINC-1)/C-X-C motif chemokine ligand 1 (CXCL1) [88,89,90]. In addition to increasing the vascular permeability of brain microvessels, these chemokines serve as chemoattractants that lead to blood inflammatory cells in the brain. The production of astrocytic MCP-1/CCL2 and CINC-1/CXCL1 is increased by the stimulation of ET_B_-R [21,90]. In contrast, stimulation of ET_B_-R decreased the expression of astrocytic ANG-1 and SHH [31,32], which are factors that enhance the integrity of brain microvessels and protect the BBB from brain injury [31,32,86]. These actions of ET-1 on vascular permeability-regulating factors suggest that the activation of astrocytic ET_B_-R signaling impairs BBB function, resulting in brain edema and neuroinflammation.

As described above, astrocytes also play a role in supporting neuroprotection and repair of damaged nerve systems. Stimulation of ET_B_-R increases the production of factors promoting neuroprotection/neuronal regeneration, including glial cell line-derived neurotrophic factor (GDNF), BDNF, NT-3, NGF, and basic fibroblast growth factor (bFGF) [91,92,93]. Ephrin is a membrane-associated protein family that regulates adhesion-dependent cellular functions by interacting with Eph receptors [94]. Several ephrin subtypes are expressed in astrocytes. Increased expression of astrocytic ephrins in brain disorders inhibited axon elongation and synaptogenesis during the regeneration of damaged nerve tissues [95,96,97,98,99,100]. The expression of ephrin-A2, -A4, -B2, and -B3 in cultured astrocytes was decreased by stimulation of ET_B_-R [101]. Both the increase in neurotrophic factors and the decrease in ephrins suggest that stimulation of astrocytic ET_B_ receptors plays a role in promoting neuroprotection/neuronal regeneration of damaged nerve tissues.

## 5. Roles of Astrocytic ET_B_-R in the Acute Phase of TBI

Under normal conditions, the function of the BBB is maintained by a balance between astrocyte-derived factors that increase vascular permeability and enhance the integrity of brain microvascular endothelial cells. In the acute phase of TBI, the production of astrocytic factors that enhance vascular permeability causes BBB disruption in the injured areas, which results in brain edema and neuroinflammation. ET-1 is upregulated in the acute phase of TBI and regulates the production of these astrocytic factors through ET_B_-R [13]. Therefore, we investigated the effects of ET_B_-R antagonists on BBB disruption in fluid percussion injury (FPI)-induced TBI model mice [13]. In TBI model mice, BBB permeability was remarkably increased one to five days after FPI. Furthermore, an increased expression level of ET_B_-R and proliferation of reactive astrocytes were also observed [13,56]. Thus, proliferation signal pathways of astrocytic ET_B_-R are activated and promote BBB permeability in TBI. Intracerebroventricular administration of the ET_B_-R antagonist BQ788 from 2 to 5 days after FPI significantly reduced TBI-induced BBB disruption and brain edema in the acute phase (three to five days after FPI), accompanied by a reduction in the number of GFAP-positive reactive astrocytes [13,31,32]. Thus, an ET_B_-R antagonist inhibits excessive proliferation signal pathways of astrocytic ET_B_-R, resulting in the reduction in BBB disruption and brain edema. On the other hand, administration of the ET_A_-R antagonist FR139317 from 2 to 5 days after FPI had no significant effects on BBB disruption and increased reactive astrocytes [13]. Guo et al. [102] found that BQ788 reduced neutrophils and monocytes and decreased the expression levels of cytokines and inflammatory mediators in the spinal cord after contusion injury. Although no selective ET_B_ receptor antagonists, including BQ788, have been clinically applied, some ET antagonists have been used as therapeutics for pulmonary arterial hypertension [49]. We also found that the intravenous administration of bosentan, a non-selective ET antagonist, reduced FPI-induced BBB disruption and brain edema [56]. These results indicate that antagonism of ET_B_-R during the acute phase of TBI is effective in protecting the BBB.

**Figure 5:**
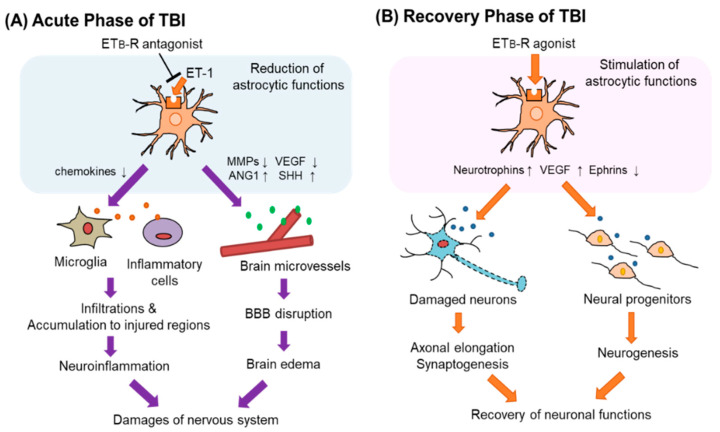
Astrocytic ET_B_-R as a therapeutic target of TBI. Astrocytic ET_B_-R may have different significance as a therapeutic target for patients with TBI in the acute and recovery phases. (**A**) In acute TBI, increased ET-1 increases the expression of MMPs and VEGF-A through the astrocytic ET_B_-R. It reduces angiopoietin-1 (ANG-1) and sonic hedgehog (SHH) production. Altered production of these vascular permeability regulators causes BBB disruption that induces brain edema. Stimulation of ET_B_-Rs also attracts inflammatory cells to the site of injury through increased production of chemokines. Administration of ET_B_-R antagonists in the acute phase of TBI reduces these astrocytic functions and may protect the nervous system. (**B**) In the recovery phase of TBI, the administration of ET_B_-R agonists is expected to be effective in recovering neuronal function impaired by TBI. Stimulation of astrocytic ET_B_-Rs promotes the production of neurotrophic factors, which promotes synaptogenesis and neurogenesis. ET_B_-R agonists also reduce the expression of ephrins. Then, regeneration of the damaged nervous system is promoted.

BBB protection by ET_B_-R antagonists is mediated by the normalization of the expression of astrocyte-derived vascular permeability regulators. TBI increased the expression levels of MMP-9 and VEGF-A in reactive astrocytes [13]. Administration of BQ788 and bosentan significantly decreased the expression of MMP-9 and VEGF-A in FPI-induced TBI model mice [13,56]. On the other hand, expression levels of astrocytic ANG-1 and SHH, which were increased by TBI, were further increased by the administration of BQ788 and bosentan [31,32,56]. Supporting the results in a TBI animal model, we confirmed that the altered expression of these permeability regulators by ET-1 was inhibited by BQ788 in cultured astrocytes [31,32,56]. These results imply that antagonism of ET_B_-R in the acute phase of TBI can alleviate BBB disruption by controlling the expression of astrocyte-derived bioactive factors (Figure 5A).

## 6. Roles of Astrocytic ET_B_-R in the Recovery Phase of TBI

Neuroinflammation and cerebral ischemia in the acute phase of TBI induce neuronal degradation in injured areas, leading to impairment of motor, cognitive, and emotional functions in patients with TBI [1,2]. To improve these nerve dysfunctions, it is important to reduce damage in the acute phase of TBI and promote neurogenesis and synaptic regeneration in the recovery phase, which is conventionally performed by physical therapy. The direct application of several neurotrophic factors was initially trialed to recover nerve dysfunction in patients with brain insults and neurodegenerative diseases. However, owing to the difficulty of intracerebral delivery and the occurrence of side effects, none of them has been clinically used [5,103]. Recently, a clinical trial of genetically modified bone marrow-derived mesenchymal stem cells (SB623) in patients with TBI in the recovery phase was conducted to examine the effectiveness of recovery of impaired nerve functions [104]. However, no low molecular weight drugs used for peripheral administration have been clinically applied, and a new therapeutic strategy is desired.

Reactive astrocytes produce many factors that regulate the regeneration of the nervous system, including neurotrophic factors and cell adhesion molecules [105,106]. Regulating the production of astrocyte-derived factors is proposed as an effective approach to promote the regeneration of the damaged nervous system [107]. Some astrocyte-derived factors have been shown to play roles in nerve regeneration after TBI [108,109], suggesting that impaired nerve function can be recovered by regulating astrocytic function. ET_B_-R signaling stimulates the production of neurotrophic factors, including NGF, bFGF, BDNF, NT-3, and VEGF, [83,84,85,91,92,93]. In addition, ET_B_-R signaling decreased the expression of ephrins, which are negative regulators of neurite outgrowth and neurogenesis in the repair of damaged nerve systems [101]. The effects of ET_B_-R signaling on the expression of trophic factors and ephrins suggest that stimulation of astrocytic ET_B_-R promotes the regeneration of the nervous system. In TBI model mice, we found that increased expression of ET_B_-R and reactive astrocytes were also observed not only in the acute phase but also in the recovery phase (seven days after FPI) [13,56]. Thus, the proliferation signal pathway of astrocytic ET_B_-R is also activated in the recovery phase and enhances the production of astrocytic neurotrophic factors. In addition, ET-1 may affect neural progenitor cells to promote the regeneration of the nervous system through ET_B_-R. During the development of the enteric nervous system, the proliferation and migration of neural progenitor cells are regulated by the ET-3/ET_B_-R signal [110,111]. Nishikawa et al. observed in an ex vivo culture system of mouse cortical tissue that an ET_B_-R agonist, IRL-1620 (sovateltide), enhanced interkinetic nuclear migration of cerebral neural progenitor cells, whereas BQ788, an ET_B_-R antagonist, reduced it [112]. This report also showed that BQ788 decreased the number of newborn neurons. Although the role of ET_B_-R in nerve regeneration after TBI has not been investigated, some studies in animal models have suggested that stimulation of ET_B_-R promotes regeneration of the damaged nervous system. Leonard et al. [113,114] showed that intravenous treatment with IRL-1620 enhanced angiogenesis and neurogenesis and reduced neurological damage in a rat model of permanent cerebral ischemia. Briyal et al. [115] suggested that IRL-1620 prevents beta-amyloid-induced oxidative stress and cognitive impairment in rats. These findings raise the possibility that stimulation of ET_B_-R during the recovery phase of TBI also has a beneficial effect on the improvement of impaired nerve function (Figure 5B).

## 7. Significance of Astrocytic ET_B_-R as a Therapeutic Target of TBI

In this article, we have discussed the importance of ET_B_-R signaling in regulating the pathophysiological functions of astrocytes, which show both detrimental and beneficial effects on the nervous system in response to brain disorders. Both astrocytic actions were promoted by stimulation with ET_B_-R. This indicates that the selective use of ET_B_-R antagonists/agonists according to the pathological course of TBI is necessary to obtain therapeutic benefits. In the acute phase of TBI, the increased production of ET-1 induces BBB disruption and brain edema through astrocytic ET_B_-R. Therefore, ET_B_-R antagonists may be effective in reducing secondary damage. Some drugs with ET_B_-R antagonistic activity, such as bosentan and macitentan, have been clinically applied for pulmonary hypertension [49]. These ET antagonists are expected to expand their clinical application to protect against nerve damage during the acute phase of TBI. In contrast, stimulation of astrocytic ET_B_-R increases the production of several neurotrophic factors and decreases the expression of ephrin subtypes. Because these ET_B_-R-mediated functional alterations of astrocytes can promote regeneration of the impaired nervous system, the application of ET_B_-R agonists may show beneficial effects in patients with TBI during the recovery phase. In clinical trials of selective ET_B_ agonists, IRL-1620 has been evaluated in patients with acute cerebral ischemic stroke [116].

Despite societal demands, no effective drug treatments are currently in clinical use for patients with TBI. In addition, targeting drugs for astrocytes have not been developed although multiple studies suggest that astrocytes play key roles in the pathogenesis of TBI. Because astrocytic ET_B_-R signaling is closely involved in the pathogenesis of TBI and regulates astrocyte functions including activation, proliferation, and production of bioactive factors, targeting drugs for astrocytic ET_B_-R may resolve two problems at the same time. Although further preclinical verification is required regarding its efficacy, astrocytic ET_B_-R is expected to be a novel target for drugs that aim to protect/improve brain functions impaired by TBI.

## Data Availability

Not applicable.
